# Targetless LiDAR–camera extrinsic calibration via semantic distribution alignment

**DOI:** 10.3389/frobt.2026.1760867

**Published:** 2026-03-09

**Authors:** Xi Chen, Bingyu Sun

**Affiliations:** 1 Hefei Institutes of Physical Science, Chinese Academy of Sciences, Hefei, China; 2 University of Science and Technology of China, Hefei, China

**Keywords:** directional observability weighting, Jensen–Shannon divergence, robotic perception, semantic distribution alignment, targetless LiDAR–camera calibration

## Abstract

**Introduction:**

LiDAR–camera fusion systems are widely used in robotic localization and perception, where accurate extrinsic calibration is crucial for multi-sensor fusion. During long-term operation, extrinsic parameters can drift due to vibration and other disturbances, while target-based recalibration is inconvenient in the field and targetless approaches often suffer from highly non-convex objectives and limited robustness in challenging outdoor scenes.

**Methods:**

We propose a targetless LiDAR–camera extrinsic calibration method by minimizing a semantic distribution consistency risk on SE(3). We align semantic probability distributions from the two sensing modalities in the image domain and freeze the pixel sampling measure at an anchor pose, so that pixel weighting no longer depends on the current extrinsic estimate and the objective landscape remains stable during optimization. On top of this anchor-fixed measure, we introduce a direction-aware weighting strategy that emphasizes pixels sensitive to yaw perturbations, improving the conditioning of rotation estimation. We further use a globally balanced Jensen–Shannon divergence to mitigate semantic class imbalance and enhance robustness.

**Results:**

Experiments on the KITTI Odometry dataset show that the proposed method reliably converges from substantial initial perturbations and yields stable extrinsic estimates.

**Discussion:**

The results indicate that the method is promising for maintaining long-term LiDAR–camera calibration in real-world robotic systems.

## 1 Introduction

Perception and localization in mobile robots and autonomous vehicles increasingly rely on multi-sensor fusion. LiDAR offers accurate three-dimensional range measurements, while cameras provide rich appearance information and strong semantic cues. However, effective multi-sensor fusion depends strongly on accurate extrinsic parameters. In most practical workflows, these parameters are calibrated manually or semi-automatically in an offline procedure before the robot or vehicle is built or first deployed. However, during long-term robot operation, factors such as mechanical vibration, load variations, and external disturbances can gradually shift the pre-calibrated extrinsic parameters. Even modest drift can weaken cross-modal alignment and, as a result, degrade perception and localization performance. These considerations underscore the value of developing a targetless calibration method that remains accurate and dependable in real operating environments.

Although a number of extrinsic calibration methods have been proposed, most still fall short when it comes to performing targetless recalibration in the kinds of conditions robots actually operate in. Broadly speaking, existing LiDAR–camera calibration techniques split into target-based procedures and targetless approaches. Target-based methods use dedicated patterns and controlled environments to achieve high accuracy and good repeatability (Zhang and Pless, 2004; Geiger et al., 2012; An et al., 2020; Cai et al., 2020), but they are cumbersome to deploy during routine operation. Targetless methods exploit natural scene structure, motion, or semantic cues instead of artificial targets (Moghadam et al., 2013; Pandey et al., 2012; Taylor et al., 2013; Koide et al., 2023; Ma et al., 2021; Strobl and Hirzinger, 2006; Ishikawa et al., 2018). However, many of these formulations still suffer from non-convex and sometimes effectively iteration-dependent objectives (e.g., when associations, sampling sets, or pixel-wise weights are updated with the current pose), sensitivity to initialization and motion excitation, and degraded performance in low-excitation scenarios (see Section 2 for a detailed review).

More recently, semantic-consistency-based targetless calibration methods (e.g., aligning semantic edges or object masks) have attempted to leverage high-level semantic information to mitigate low-level geometric mismatches (Liao et al., 2023; Luo et al., 2024). However, directly aligning discrete labels or masks introduces strong non-convexity and makes the methods sensitive to segmentation noise and dynamic occlusions. Furthermore, updating pixel weights together with extrinsics may produce a “moving yardstick” effect that reduces numerical stability.

To address these issues, we cast LiDAR–camera extrinsic calibration as minimizing a distributional inconsistency risk over rigid-body transformations on the SE(3) manifold. Instead of aligning sparse correspondences or discrete labels, we align continuous, pixel-wise *semantic probability fields* in the image domain: the camera provides per-pixel semantic probabilities, while semantically labeled LiDAR points are projected into the image and softly aggregated into a dense LiDAR-induced semantic probability field. The extrinsic parameters are then estimated by minimizing a robust distributional divergence between the two fields, augmented with a global semantic-class histogram consistency term.

To improve stability and identifiability during optimization, we introduce three design strategies: (1) *anchor-fixed sampling*, where the pixel sampling/weighting is computed once at an anchor estimate and kept fixed within each optimization stage to avoid the “moving yardstick” effect; (2) *direction-aware (yaw-sensitive) weighting*, which emphasizes pixels whose LiDAR-induced semantics change most under small yaw perturbations to improve conditioning for rotation estimation in road-dominant scenes; and (3) a *robust objective* based on a log-saturated Jensen–Shannon divergence together with global histogram balancing and a multi-scale organization to mitigate outliers, noise, and class imbalance. Formal definitions of all symbols and operators are provided in Section 3.1–3.4. The complete mathematical formulation is provided in Equations 1–26.

In summary, the proposed method aims to maintain stable extrinsic estimates during long-term operation and improves robustness compared with existing targetless calibration objectives. Our main contributions are as follows:

1. We propose a targetless LiDAR–camera extrinsic calibration framework that aligns dense semantic probability fields in the image domain and optimizes the extrinsic parameters directly on SE(3) .
2. We introduce an anchor-fixed sampling strategy that decouples pixel sampling/weighting from the evolving pose estimate, improving numerical stability by avoiding measure/metric drift during iterative optimization.
3. We design a direction-aware yaw observability weighting that emphasizes pixels most informative for yaw estimation, improving identifiability in road-dominant or low-structure scenes.
4. We develop a robust objective based on a log-saturated Jensen–Shannon divergence with global semantic histogram balancing and a multi-scale formulation, and solve it using a Riemannian Gauss–Newton optimizer. We validate the method on public driving datasets under controlled initialization biases.

The remainder of this paper is organized as follows. Section 2 reviews target-based, targetless, and semantic-driven calibration methods. Section 3 describes the proposed approach in detail. Section 4 presents the experimental setup, baseline comparisons, factor analysis, and ablation studies. Section 6 concludes the paper and discusses possible future directions.

## 2 Related work

LiDAR–camera extrinsic calibration methods can be broadly grouped into target-based approaches and targetless approaches. Within the targetless category, semantic-driven methods have recently emerged as an important subclass. Although target-based and targetless methods have established relatively mature offline calibration procedures, they often face challenges related to adaptability and robustness in continuous or long-term operation. In recent years, semantic-consistency-based approaches have attempted to alleviate low-level geometric mismatches by leveraging high-level semantic information. Several survey papers have systematically summarized these developments and highlighted remaining open challenges (Li et al., 2023; An et al., 2024).

### 2.1 Target-based methods

Target-based methods rely on predefined geometric patterns to establish accurate 3D–2D correspondences and solve for extrinsic parameters using reprojection or geometric constraints. Early work largely focused on calibrating a camera with a 2D laser range finder (2D LiDAR). Zhang and Pless used a planar checkerboard target and point-on-plane constraints (Zhang and Pless, 2004), whereas Chen et al. employed a cubic calibration target covered with chessboard patterns and derived point-to-line constraints for 2D LiDAR–camera calibration (Chen et al., 2012). These formulations led to a large body of follow-up work on target design, geometric modeling, and nonlinear refinement.

To reduce manual effort, Geiger et al. later proposed a single-shot method that jointly calibrates cameras and range sensors (Geiger et al., 2012). More recent studies have designed fiducial patterns specifically adapted to modern solid-state LiDARs (Xie et al., 2022). Recent work further improves plane-based target calibration by modeling the uncertainty of checkerboard planes and constructing analytic plane covariances, which enables principled weighting in the optimization (Koo et al., 2020).

While target-based approaches can deliver high accuracy and good repeatability in controlled conditions, they fundamentally rely on dedicated calibration objects and carefully arranged scenes. In practice, this means that calibration often has to be performed in a separate, instrumented environment, limiting the usefulness of these methods for continuous deployment and long-term operation, where systems are expected to maintain calibration without access to such controlled setups (Li et al., 2023; An et al., 2024).

### 2.2 Targetless methods

Compared with target-based approaches, targetless calibration does not rely on artificial calibration objects and is therefore better suited for recalibration in real operating environments. Existing targetless methods can be broadly grouped into two categories: geometric or information-theoretic alignment, and motion-based approaches.

#### 2.2.1 Geometric and information-theoretic methods

Geometric alignment methods construct cross-modal correspondences by leveraging naturally occurring environmental structures—such as planes, edges, and corners—and solve for the extrinsics by minimizing geometric consistency error. Representative methods use Gaussian mixture models and coarse-to-fine optimization procedures, achieving good performance in structured scenes; however, they remain sensitive to feature detection quality and initialization (Castorena et al., 2016; Kang and Doh, 2020; Yin et al., 2023). Information-theoretic approaches maximize the statistical dependence between sensor modalities without performing explicit feature matching. Pandey et al. introduced a mutual-information-based method (Pandey et al., 2012). Although broadly applicable, such methods often produce non-convex cost landscapes with multiple local optima, making them highly sensitive to initialization and sometimes requiring global search strategies. More recently, Koide et al. developed a general single-shot toolbox for targetless calibration based on MI and NMI (Koide et al., 2023).

#### 2.2.2 Motion-based methods

Motion-based methods infer extrinsic parameters by analyzing relative sensor motions across multiple time steps, typically cast as a hand–eye calibration problem (Strobl and Hirzinger, 2006). While hand–eye calibration itself does not require overlapping sensor fields of view, some LiDAR–camera pipelines use it only for initialization and then perform a correspondence-based refinement that does require overlap (e.g., Ishikawa et al., 2018).

However, these methods strongly depend on motion estimation quality and require sufficient excitation, and they are particularly sensitive to sensor synchronization errors and low-excitation conditions such as near-straight-line trajectories.

### 2.3 Semantic-driven targetless methods

Semantic-driven calibration approaches form a subclass of targetless methods and make use of high-level semantic cues such as categories, instances, or semantic edges to improve robustness in targetless and online scenarios. Tsaregorodtsev et al. incorporated semantic segmentation of images and point clouds to define registration objectives tailored for infrastructure–vehicle configurations (Tsaregorodtsev et al., 2022). SE-Calib introduced a semantic-edge consistency objective for online boresight calibration under urban environments (Liao et al., 2023). Calib-Anything used the Segment Anything Model (SAM) to generate zero-shot semantic masks and optimized cross-modal consistency across semantic object regions (Luo et al., 2024). Other semantic-based methods include semantic mutual information estimation (SemCal) Jiang et al. (2021), consistency-based single-shot inference (RobustCalib) (Xu et al., 2023), and semantic alignment for online calibration in autonomous driving (Zhu et al., 2020). However, semantic-driven approaches often directly align discrete labels or masks, resulting in strongly non-convex optimization landscapes and high sensitivity to segmentation noise and dynamic occlusions, as also emphasized in recent surveys (Li et al., 2023; An et al., 2024). From a numerical perspective, jointly recomputing pixel or mask weights and updating the extrinsic parameters effectively changes the underlying measurement reference during optimization, which can lead to a “moving yardstick” effect and reduce stability in practice.

In summary, existing target-based and targetless approaches, including recent semantic-driven variants, have advanced LiDAR–camera calibration but still struggle to maintain stable optimization and to prevent extrinsic drift in changing environments. We propose a unified semantic-consistency framework that combines an anchor-fixed sampling measure, direction-aware yaw weighting, and a robust objective on semantic probability fields, aiming for more reliable calibration in realistic conditions and better support for long-term deployment.

## 3 Proposed methods

**FIGURE 1:**
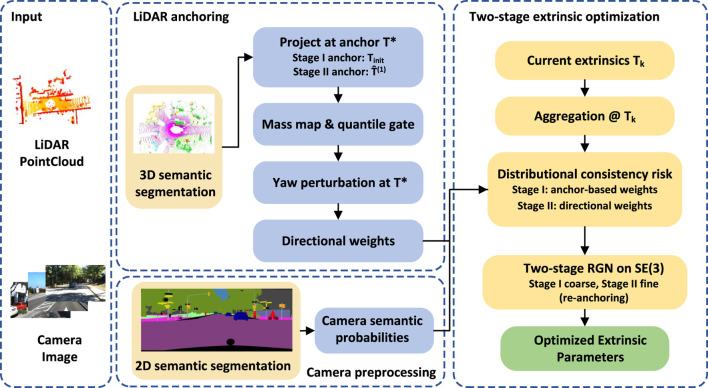
Schematic pipeline of the proposed LiDAR–camera extrinsic calibration method via semantic distribution alignment on SE(3) .

We formulate LiDAR–camera extrinsic calibration as a distributional consistency risk minimization problem on the special Euclidean group SE(3) . Concretely, we represent the camera output as a semantic probability field P(u) in the image domain and construct a LiDAR-induced field $Q_{T}\left(u\right)$ by projecting semantically labeled LiDAR points into the image via an aggregation operator. To avoid metric drift during optimization, we freeze the pixel sampling measure at an anchor pose $T^{⋆}$ and build yaw-sensitive pixel weights $w^{\left(\ell \right)}\left(u\right)$ at each scale ℓ by combining this fixed measure with a direction-aware observability weighting. On top of these fields, we define a log-saturated distributional consistency objective L(T) based on multi-scale Jensen–Shannon (JS) divergence, complemented by global semantic histogram balancing to mitigate class imbalance. The resulting objective is minimized on SE(3) using a Riemannian Gauss–Newton method with Levenberg–Marquardt damping, embedded in a two-stage scheme: a coarse stage without yaw weighting to pull the estimate into a good basin, followed by a fine stage that reintroduces directional weighting to refine the yaw and translation. A schematic overview of the full pipeline is shown in Figure 1.

### 3.1 Problem setup and notation

Let the image domain be Ω={1,…,W}×{1,…,H},u∈Ω, and let the number of semantic classes be C with class index c∈{1,…,C} . The camera-side semantic probability field is written as

$$P\;:\;\Omega \to \Delta^{C-1},\;\overset{C}{\underset{c=1}{\sum }}P\left(u,c\right)=1,$$ (1)

where $\Delta^{C-1}$ denotes the (C−1) -dimensional probability simplex over C classes.

The LiDAR sensor provides a set of semantically labeled points in its own coordinate frame, ${\left\{\left(X_{i},y_{i}\right)\right\}}_{i=1}^{N},\;X_{i}\in R^{3},\;y_{i}\in \left\{1,\ldots ,C\right\}.$ The extrinsic parameters are denoted by T=(R,t)∈SE(3) , where R∈SO(3) and $t\in R^{3}$ . The pinhole projection operator is $\pi \;:\;R^{3}\to R^{2}$ . In the implementation, we undistort the images in advance so that π depends only on the intrinsic matrix K . After transforming LiDAR points into the camera frame as ${\tilde{X}}_{i}=TX_{i}$ , points with depth Z≤0 (we use a more conservative threshold Z≤0.1 m in practice) or whose projections fall outside the image bounds are discarded.

To construct a smooth semantic field from sparse LiDAR points, we use a truncated Gaussian kernel

$$\Gamma \left(u;\hat{u}\right)=\exp\;\left(-\frac{\|u-\hat{u}{\|}_{2}^{2}}{2\sigma^{2}}\right)\;1_{\left\{\|u-\hat{u}{\|}_{2}\leq 3\sigma \right\}},\;\sigma =1.0\text{ px}.$$ (2)

With this kernel, each pixel aggregates only a finite and spatially local set of projected points, which limits the influence of far-away measurements and stabilizes the constructed semantic field. In practice, the resulting objective L(T) is sufficiently smooth for second-order methods such as Gauss–Newton to behave robustly in our experiments.

### 3.2 Semantic field and aggregation operator $A_{T}$

Given a candidate extrinsic T , we first transform LiDAR points from the LiDAR frame to the camera frame, ${\tilde{X}}_{i}=TX_{i}$ , and then project them onto the image plane using π . Only points with positive depth and projections within [1,W]×[1,H] are kept for aggregation.

At pixel u , the unnormalized semantic mass of class c is defined as

$${\tilde{Q}}_{T}\left(u,c\right)=\underset{i:\;y_{i}=c}{\sum }\Gamma \left(u;\pi \left(TX_{i}\right)\right).$$ (3)

After normalization, we obtain the LiDAR-induced semantic probability field in the image domain,

$$Q_{T}\left(u,c\right)=\frac{{\tilde{Q}}_{T}\left(u,c\right)+\varepsilon /C}{\sum_{k=1}^{C}{\tilde{Q}}_{T}\left(u,k\right)+\varepsilon },\;\varepsilon =10^{-8}.$$ (4)

Equations 3, 4 define an aggregation operator $A_{T}$ that maps the semantic point set ${\left\{\left(X_{i},y_{i}\right)\right\}}_{i=1}^{N}$ to the semantic field $Q_{T}$ ,

$$A_{T}\left({\left\{\left(X_{i},y_{i}\right)\right\}}_{i=1}^{N}\right)=Q_{T}.$$ (5)

To avoid numerical underflow, we truncate entries of P(u,⋅) and $Q_{T}\left(u,\cdot \right)$ that are smaller than ε and renormalize them, so that the resulting vectors still lie in the simplex $\Delta^{C-1}$ .

To improve robustness to scale changes and noise, we construct semantic fields at two resolutions: the original resolution ℓ=1.0 and a half resolution ℓ=0.5 . After obtaining P and $Q_{T}$ at the original resolution, we apply Gaussian smoothing and downsampling to $S\in \left\{P,Q_{T}\right\}$ : $S^{\left(0.5\right)}={Down}_{0.5}^{\text{bilinear}}\left({Gauss}_{1.6}\left(S\right)\right)$ , $\;S^{\left(1.0\right)}={Gauss}_{1.3}\left(S\right),$ and then apply the same clamping and renormalization at each scale so that $P^{\left(\ell \right)}\left(u,\cdot \right),\;Q_{T}^{\left(\ell \right)}\left(u,\cdot \right)\in \Delta^{C-1}$ for ℓ∈{0.5,1.0} .

### 3.3 Anchor-fixed measure and directional observability weighting

If pixel weights depend directly on the current pose T , then the “ruler” of the objective changes whenever T changes during optimization. This can distort the loss landscape and weaken yaw observability. To avoid this, we freeze the sampling measure at an anchor pose $T^{⋆}$ and then place a yaw-sensitivity weighting on top of it.

At the beginning of a local optimization stage, we set the anchor as $T^{⋆}:=T_{\text{init}},$ where $T_{\text{init}}$ comes from the previous estimate or from an external initialization. Within this stage, $T^{⋆}$ remains fixed. From the definition in (3) at the anchor pose, we obtain the unnormalized semantic mass ${\tilde{Q}}_{T^{⋆}}$ .

To steer the sampling measure toward structurally informative regions, we introduce a non-road mass weight $\lambda_{\text{nr}}\geq 0$ and split the class index set as follows: $C=\left\{1,\ldots ,C\right\},\;C_{\text{bg}}\subset C\;\left(\text{e.g., road, sidewalk, sky}\right),\;C_{\text{nr}}=C\backslash C_{\text{bg}}.$ The anchor-space semantic mass of non-road and background classes are then

$$M_{\text{nr}}^{⋆}\left(u\right)=\underset{c\in C_{\text{nr}}}{\sum }{\tilde{Q}}_{T^{⋆}}\left(u,c\right),\;M_{\text{bg}}^{⋆}\left(u\right)=\underset{c\in C_{\text{bg}}}{\sum }{\tilde{Q}}_{T^{⋆}}\left(u,c\right).$$ (6)

The total semantic mass map at the anchor is

$$M^{⋆}\left(u\right)=\lambda_{\text{nr}}\;M_{\text{nr}}^{⋆}\left(u\right)+M_{\text{bg}}^{⋆}\left(u\right).$$ (7)

Here $\lambda_{\text{nr}}>1$ emphasizes non-road regions such as buildings and vehicles, while $\lambda_{\text{nr}}<1$ downweights potentially noisy non-road evidence. In our default configuration, we adopt a slightly conservative setting $\lambda_{\text{nr}}=0.8$ , which empirically yields more stable calibration in sparse scenes.

Let $Q_{0.30}\left(M^{⋆}\right)$ and $Q_{0.90}\left(M^{⋆}\right)$ denote the 30th and 90th pixel-wise quantiles of $M^{⋆}\left(u\right)$ . We set $\tau_{\text{lo}}=Q_{0.30}\left(M^{⋆}\right),\tau_{\text{hi}}=Q_{0.90}\left(M^{⋆}\right),$ and construct a piecewise linear gating function

$$\phi \left(x\right)=\left\{\begin{array}{cc} 0, & x\leq \tau_{\text{lo}},\\ \frac{x-\tau_{\text{lo}}}{\tau_{\text{hi}}-\tau_{\text{lo}}}, & \tau_{\text{lo}}<x<\tau_{\text{hi}},\\ 1, & x\geq \tau_{\text{hi}}. \end{array}\right.$$ (8)

This gives a normalized anchor measure

$$s\left(u\right)=\frac{\phi \left(M^{⋆}\left(u\right)\right)}{\sum_{v\in \Omega }\phi \left(M^{⋆}\left(v\right)\right)}.$$ (9)

If the denominator is zero, we regard the frame as degenerate and discard it. At multiple scales, we apply the same smoothing and downsampling procedure as in Sec. 3.2 to ${\tilde{Q}}_{T^{⋆}}$ , which yields scale-specific measures $s^{\left(\ell \right)}$ .

To ensure that non-road classes have sufficient coverage under this measure, we compute a coverage ratio

$$r_{\text{nr}}=\frac{\sum_{u}1\left\{\sum_{c\in C_{\text{nr}}}{\tilde{Q}}_{T^{⋆}}\left(u,c\right)>\tau_{\text{lo}}\right\}}{\sum_{u}1\left\{M^{⋆}\left(u\right)>\tau_{\text{lo}}\right\}}.$$ (10)

We require $r_{\text{nr}}\geq \rho_{\text{nr}}$ ; otherwise the frame is considered to have insufficient semantic coverage and is discarded. In our default configuration, we set $\rho_{\text{nr}}=0.10$ , so that at least 10% of the high-mass pixels must carry non-road evidence for a frame to be used in calibration.

On top of this measure, we evaluate yaw-perturbation sensitivity at the anchor. Yaw is singled out because in our road-dominant setting it is typically the most weakly conditioned rotational direction (heading), whereas roll/pitch are usually bounded and already constrained by strong vertical/ground structure. The same sensitivity-based reweighting can be applied to roll or pitch by replacing ${\hat{a}}_{\text{yaw}}$ with the corresponding generators; we focus on yaw for simplicity and because it provides the dominant empirical gain. Let ${\hat{a}}_{\text{yaw}}$ denote the unit generator of rotation around the z -axis, with scalar yaw coordinate $\theta_{y}$ . Perturbations are applied in a left-multiplicative form, $\exp\left(\pm \delta {\hat{a}}_{\text{yaw}}\right)\;T^{⋆}.$ At scale ℓ , the pixel-wise sensitivity is defined as

$$d^{\left(\ell \right)}\left(u\right)={\left\|Q_{\exp\left(+\delta {\hat{a}}_{\text{yaw}}\right)T^{⋆}}^{\left(\ell \right)}\left(u,\cdot \right)-Q_{\exp\left(-\delta {\hat{a}}_{\text{yaw}}\right)T^{⋆}}^{\left(\ell \right)}\left(u,\cdot \right)\right\|}_{1},$$ (11)

and we compute the weighted mean using $s^{\left(\ell \right)}$ ,

$${\bar{d}}^{\left(\ell \right)}=\underset{u}{\sum }s^{\left(\ell \right)}\left(u\right)\;d^{\left(\ell \right)}\left(u\right).$$ (12)

In our implementation, we use fixed parameters β=2 and δ=0.1° . The final pixel weights at scale ℓ are

$$w^{\left(\ell \right)}\left(u\right)=\frac{s^{\left(\ell \right)}\left(u\right){\left(d^{\left(\ell \right)}\left(u\right)/{\bar{d}}^{\left(\ell \right)}\right)}^{\beta }}{\sum_{v}s^{\left(\ell \right)}\left(v\right){\left(d^{\left(\ell \right)}\left(v\right)/{\bar{d}}^{\left(\ell \right)}\right)}^{\beta }},$$ (13)

At full resolution, we write $w\left(u\right)≜w^{\left(1.0\right)}\left(u\right)$ .

Intuitively, $d^{\left(\ell \right)}\left(u\right)$ approximates the first-order sensitivity $\partial_{\theta_{y}}Q^{\left(\ell \right)}\left(u,\cdot \right)$ at pixel u , and its magnitude is monotonically related to a Fisher-information-like term along yaw. Reweighting based on $d^{\left(\ell \right)}\left(u\right)$ therefore acts as a numerical pre-conditioning that strengthens the yaw component of the local (Gauss–Newton) Hessian, increasing the curvature with respect to $\theta_{y}$ and facilitating yaw correction in practice. Since rotational and translational increments are coupled by the projection model in the same normal equations, improving the yaw curvature also tends to improve the conditioning of the full 6-DOF update, mitigating yaw–translation ambiguity.

### 3.4 Distributional consistency risk

We use a log-saturated robust function

$$\psi_{\tau }\left(z\right)=\tau \log\left(1+z/\tau \right),\;\tau =0.1,$$ (14)

and measure consistency between semantic distributions using the Jensen–Shannon (JS) divergence

$$JS\left(p,q\right)=\frac{1}{2}KL\left(p\|m\right)+\frac{1}{2}KL\left(q\|m\right),\;m=\frac{p+q}{2},$$ (15)

where the Kullback–Leibler divergence uses the natural logarithm. For discrete distributions we have JS(p,q)∈[0,ln⁡2] , so $\psi_{\tau }$ remains smooth and bounded over the full range.

At scale ℓ , the pixel-wise distributional consistency term is

$$E^{\left(\ell \right)}\left(T\right)=\underset{u\in \Omega^{\left(\ell \right)}}{\sum }w^{\left(\ell \right)}\left(u\right)\;\psi_{\tau }\;\left(JS\left(P^{\left(\ell \right)}\left(u\right),Q_{T}^{\left(\ell \right)}\left(u\right)\right)\right).$$ (16)

To reduce the effect of class imbalance, we add a global histogram term at full resolution. Using the weights w(u) , we define

$$h_{P}\left(c\right)=\underset{u\in \Omega }{\sum }w\left(u\right)\;P\left(u,c\right),\;h_{Q}\left(c\right)=\underset{u\in \Omega }{\sum }w\left(u\right)\;Q_{T}\left(u,c\right),$$ (17)

and construct

$$H\left(T\right)=\psi_{\tau }\left(JS\left(h_{P},h_{Q}\right)\right).$$ (18)

The final unified calibration objective is

$$L\left(T\right)=E^{\left(0.5\right)}\left(T\right)+E^{\left(1.0\right)}\left(T\right)+H\left(T\right).$$ (19)

Because each $w^{\left(\ell \right)}$ is a probability measure and JS∈[0,ln⁡2] with $\psi_{\tau }$ monotone and bounded, these three terms naturally stay on a similar scale. In practice, we do not introduce extra manual weights between them.

### 3.5 Yaw observability analysis

In this subsection, $\partial_{\theta_{y}}$ denotes the directional derivative associated with a left perturbation $\exp\left(\theta_{y}{\hat{a}}_{\text{yaw}}\right)$ . We assume that the truncated Gaussian kernel and any depth-based weights satisfy basic boundedness conditions, and that there exist constants $\epsilon_{p},\epsilon_{q}>0$ such that, after truncation and normalization,

$$\underset{u,c}{\min}P\left(u,c\right)\geq \epsilon_{p},\;\underset{u,c}{\min}Q_{T_{◦}}\left(u,c\right)\geq \epsilon_{q},$$ (20)

where $T_{◦}$ is the true extrinsic. We let ν be the pixel sampling measure induced by w(u) .

If there exists a constant κ>0 such that

$$\underset{u\in \Omega }{\sum }w\left(u\right)\;{\left\|\partial_{\theta_{y}}Q_{T_{◦}}\left(u,\cdot \right)\right\|}_{1}^{2}\geq \kappa ,$$ (21)

then we say that $\left(P,Q_{T_{◦}},\nu \right)$ is observable in the yaw direction. Intuitively, (21) requires that a small yaw perturbation induces a non-negligible change in the LiDAR-induced semantic distribution under the sampling measure ν .

Under the regularity assumptions in (20), the JS divergence is strongly convex in both arguments on the interior of the simplex, and its Hessian admits a uniform lower bound that depends only on $\epsilon_{p}$ and $\epsilon_{q}$ . Combining this strong convexity with (21) and the fact that w(u) defines a probability measure, one can show that there exists a constant η>0 such that, in a neighborhood of $T_{◦}$ ,

$$\frac{\partial^{2}}{\partial \theta_{y}^{2}}L\left(T_{◦}\right)\geq \eta \kappa .$$ (22)

A full derivation is beyond the scope of this paper; the role of (22) here is to provide an analytical motivation for the yaw-weighting scheme.

For simplicity, we state the result in terms of the full-resolution term; the additional multi-scale and histogram terms are non-negative and share the same sampling measure, so they can only increase the curvature of L(T) along the yaw direction. In other words, the yaw observability condition implies a strictly positive local lower bound on the second derivative of L(T) along the yaw direction, which helps parameter identification and supports stable convergence of second-order optimization methods.

### 3.6 Riemannian Gauss–Newton on SE(3) and two-stage optimization

To minimize L(T) , we rewrite it as a multi-scale weighted nonlinear least-squares problem and solve it on SE(3) using a Riemannian Gauss–Newton (RGN) method with Levenberg–Marquardt damping.

At each scale ℓ and pixel u , we first compute

$$z^{\left(\ell \right)}\left(u\right)=JS\left(P^{\left(\ell \right)}\left(u\right),Q_{T}^{\left(\ell \right)}\left(u\right)\right),\;z^{\left(\ell \right)}\left(u\right)\leftarrow \max\left(z^{\left(\ell \right)}\left(u\right),\varepsilon \right),$$ (23)

and use $e^{\left(\ell \right)}\left(u\right)=z^{\left(\ell \right)}\left(u\right)$ as the working residual for that pixel. The global histogram term is treated in the same way: $z_{h}=JS\left(h_{P},h_{Q}\right),z_{h}\leftarrow \max\left(z_{h},\varepsilon \right),e_{h}=z_{h}.$ Given $\psi_{\tau }^{\prime }\left(z\right)=\frac{\tau }{\tau +z}$ , we adopt an IRLS scheme and define

$$\gamma \left(z\right)≜\frac{\psi_{\tau }^{\prime }\left(z\right)}{z}=\frac{\tau }{\left(\tau +z\right)\;z}.$$ (24)

$$\alpha^{\left(\ell \right)}\left(u\right)=w^{\left(\ell \right)}\left(u\right)\;\gamma \left(z^{\left(\ell \right)}\left(u\right)\right),\;\alpha_{h}=\gamma \left(z_{h}\right).$$ (25)

With these weights, the robust objective is locally equivalent to a weighted $\ell_{2}$ norm of the residual vector e in the sense that the two formulations share the same first-order derivative with respect to T at the current iterate. This yields a standard weighted nonlinear least-squares problem amenable to a Gauss–Newton/Levenberg–Marquardt update.

Let ${\left\{{\hat{a}}_{i}\right\}}_{i=1}^{6}$ be a basis of the Lie algebra se(3) , and let $\Delta \xi \in R^{6}$ denote parameter increments in the Lie algebra. We use a left-multiplicative update on SE(3) ,

$$T_{k+1}=\exp\left(\Delta \xi_{k}\right)\;T_{k},$$ (26)

At each iteration, we stack all residuals from all scales and the histogram term into a single vector e , approximate the Jacobian $J_{e}$ along each basis direction in se(3) using central finite differences, and solve damped normal equations to obtain $\Delta \xi_{k}$ . We employ standard stopping criteria (step size, relative objective change, and maximum iterations).

Given an anchor $T^{⋆}$ and the associated multi-scale weights $w^{\left(\ell \right)}$ , the procedure above provides one local optimization of L(T) . To increase robustness to larger initialization errors, we build a two-stage optimization strategy with re-anchoring on top of this process.

#### 3.6.1 Event-driven re-anchoring

Rather than updating the anchor pose at a fixed frequency, we adopt an event-driven re-anchoring rule to keep the anchor piecewise constant during optimization. We compute $anchor\text{\_}update\text{\_}max=\max\left(\left|se3::\log\left(T_{\text{prev}}^{-1}T_{\text{new}}\right)\right|\right)$ , and re-freeze the anchor when $anchor\text{\_}update\text{\_}max>10^{-3}$ . This avoids a continuously moving reference while still preventing drift when the update becomes sufficiently large.

#### 3.6.2 Stage 1 (coarse alignment)

Given the initial extrinsic $T_{\text{init}}$ , we first set $T^{⋆}:=T_{\text{init}}$ and construct the semantic mass map $M^{⋆}\left(u\right)$ and multi-scale measures $s^{\left(\ell \right)}\left(u\right)$ as in Section 3.3. To reduce the risk of incorrect yaw weighting under large misalignment, we ignore yaw sensitivity $d^{\left(\ell \right)}\left(u\right)$ in this coarse stage and set

$${\tilde{w}}^{\left(\ell \right)}\left(u\right)=s^{\left(\ell \right)}\left(u\right).$$

When T is far from $T_{◦}$ , the finite-difference yaw perturbations $\exp\left(\pm \delta {\hat{a}}_{\text{yaw}}\right)T^{⋆}$ can project points into largely mismatched regions, so the resulting $d^{\left(\ell \right)}\left(u\right)$ may not reflect the true yaw sensitivity and can even emphasize misleading pixels. The coarse stage therefore uses only the structurally informed but direction-independent measures $s^{\left(\ell \right)}\left(u\right)$ to define a fixed sampling “ruler”. We replace $w^{\left(\ell \right)}$ with ${\tilde{w}}^{\left(\ell \right)}$ in the pixel-wise term (16) and in the IRLS weights, obtaining a direction-independent coarse objective, denoted by $\tilde{L}\left(T\right)$ . With this fixed measure, we run several RGN/LM iterations on $\tilde{L}\left(T\right)$ to move T from a larger initialization error into a local neighborhood of the true extrinsic $T_{◦}$ .

#### 3.6.3 Stage 2 (fine alignment)

After the coarse stage, we take the intermediate estimate ${\hat{T}}^{\left(1\right)}$ as a new anchor and set $T^{⋆}:={\hat{T}}^{\left(1\right)}$ . We then recompute ${\tilde{Q}}_{T^{⋆}}$ , the multi-scale measures $s^{\left(\ell \right)}\left(u\right)$ , and yaw sensitivities $d^{\left(\ell \right)}\left(u\right)$ , and from these build the full direction-aware pixel weights $w^{\left(\ell \right)}\left(u\right)$ . Under this new fixed measure, we return to the original objective L(T) in (19) and run RGN/LM until convergence to obtain the final estimate $\hat{T}$ . The directional weights $w^{\left(\ell \right)}\left(u\right)$ explicitly increase curvature along the yaw direction, which improves both the ability to correct yaw and the stability of fine optimization.

It is worth emphasizing that the two-stage strategy only introduces re-anchoring and a layered objective at the implementation level. Within each stage, the anchor $T^{⋆}$ and the corresponding weights ${\tilde{w}}^{\left(\ell \right)}$ or $w^{\left(\ell \right)}$ remain fixed, so the optimization problems reduce to minimizing $\tilde{L}\left(T\right)$ or L(T) under a fixed sampling measure.

After Stage 1, the intermediate estimate ${\hat{T}}^{\left(1\right)}$ is typically within a small neighborhood of $T_{◦}$ (see Section 4 for quantitative evidence). Since both $Q_{T}\left(\cdot \right)$ and its yaw derivative vary continuously with T , the pixel weights w(u) constructed at $T^{⋆}={\hat{T}}^{\left(1\right)}$ satisfy the yaw observability condition (21) with a possibly modified constant. Consequently, the curvature lower bound in (22) applies to the fine stage, while the coarse stage empirically enlarges the range of initial errors from which the method can still converge to a high-accuracy solution.

As with other Gauss–Newton–type calibration schemes, the proposed solver is inherently local: it leverages the curvature of L(T) only in the neighborhood of the current estimate and therefore assumes that the available initialization is not arbitrarily far from the ground truth.

#### 3.6.4 Optional coarse hypothesis initialization

When substantially larger misalignments are expected, we optionally prepend a lightweight coarse initialization step that selects a safer starting pose by evaluating a bounded set of pose hypotheses at the coarsest pyramid level. In our implementation, the hypothesis set primarily discretizes yaw within a limited range, and may additionally include a small number of translation hypotheses when translation uncertainty is non-negligible. Each hypothesis is scored using the same semantic objective L(T) (or its coarse, direction-independent variant $\tilde{L}\left(T\right)$ ), and hypotheses are evaluated independently by resetting any per-evaluation cached states before scoring so that the scores are order-independent. To avoid degenerate low-cost solutions caused by support collapse, we discard hypotheses that fail basic validity checks, such as insufficient valid frames or insufficient effective support (e.g., too few active pixels or too small accumulated weights) relative to the seed. Among the remaining hypotheses, we select a candidate primarily by objective value while discouraging solutions that reduce the cost mainly by shrinking the effective support; in addition, we retain the minimum raw-cost candidate as a conservative fallback and prefer it whenever it offers a clear cost advantage without violating the support constraints. The selected hypothesis can be optionally refined by a small local rotation micro-search and is then passed to the two-stage RGN/LM procedure as $T_{\text{init}}$ . This initialization is an optional engineering component, does not modify the underlying objective or the two-stage optimization within each stage.

## 4 Experimental results

### 4.1 Setup and evaluation protocol

#### 4.1.1 Dataset and evaluation task

Our main benchmark and the baseline comparisons in Section 4.2 are conducted on sequence 00 under the official LiDAR–camera geometric configuration. To assess cross-sequence generalization beyond a single driving sequence, we additionally evaluate our method on sequences 01 and 07 under the same protocol (Section 4.3), and evaluate robustness under network-predicted 2D/3D semantics on sequence 07 (Section 4.5).

The ground-truth extrinsics (GT) are used solely for computing evaluation metrics and for constructing a fixed semantic reference in the image domain, as described later. Importantly, GT is never introduced into the optimization process, ensuring that the optimizer always operates under unknown extrinsics and never has direct access to ground-truth information.

#### 4.1.2 Clip selection

We construct twelve fixed 50-frame clips from sequence 00, starting at frames {0, 200, 400, 600, 1,000, 2000, 2,400, 2,600, 3,000, 3,200, 3,600, 3,800}. Each clip contains 50 synchronized camera–LiDAR frames, and the same set of clips is used across all methods to ensure a fair and directly comparable evaluation. For cross-sequence generalization, we additionally sample six fixed 50-frame clips for each of sequence 01 and sequence 07, starting at frames {0, 200, 400, 600, 800, 1,000}. Unless otherwise stated, we use the same initialization bias and identical hyperparameters across all sequences.

For each clip, we run a fixed-window batch optimization: the calibrator iterates over the same set of 50 paired frames throughout optimization, without sliding the window forward in time or propagating extrinsics across windows. Consequently, accumulated calibration drift caused by online/recursive updates is not applicable under our evaluation protocol. For the scene-type analysis in Section 4.7, we additionally include several more clips to better cover different semantic compositions. Although we use fixed clips for a reproducible benchmark, we further derive a simple automatic “calibration-friendly” triggering heuristic from the scene-composition analysis in Section 4.7.

#### 4.1.3 Initialization and perturbation settings

For the main cross-method benchmark (Table 1), we use a *shared deterministic* initialization for all methods and all clips: we perturb the ground-truth (GT) extrinsics by a yaw rotation of 5° and a translation of 50 mm (expressed in the LiDAR coordinate frame). This choice ensures that every compared method starts from an identical initial estimate, avoiding confounding effects due to different random draws.

**TABLE 1:** Overall rotation and translation performance on KITTI 00 under the shared 5°/50 mm initialization. Rotation errors are in degrees; translation mean errors are in centimeters.

Method	Rot. Mean	Rot. Median	Rot. Max	Trans. Mean
Ours	0.188	0.198	0.500	0.26
CalibAnything	0.299	0.259	0.697	12.08
DirectVLCalib	5.023	5.169	10.750	8.91
Tsaregorodtsev et al.	1.595	0.936	4.688	15.85
Pandey et al. (MI)	30.822	25.852	83.950	35.74

To assess sensitivity to initialization and the practical convergence basin of our solver, we additionally run Monte-Carlo trials with *random* perturbations. In these trials, we perturb only the yaw angle (roll/pitch are kept at zero) and independently sample translations along each LiDAR axis as in Equation 27:

$$\theta_{y}∼U\left[\theta_{\min},\theta_{\max}\right],\;t_{x},t_{y},t_{z}∼U\left[t_{\min},t_{\max}\right].$$ (27)

We report results for two ranges: (i) Moderate: $\theta_{y}\in \left[-10{}^{\circ},10{}^{\circ}\right]$ and $t_{x},t_{y},t_{z}\in \left[-5,5\right]\;cm$ ; (ii) Large: $\theta_{y}\in \left[-20{}^{\circ},20{}^{\circ}\right]$ and $t_{x},t_{y},t_{z}\in \left[-10,10\right]\;cm$ . Unless otherwise stated, random-initialization experiments use the same 50-frame clips and metrics as the main benchmark.

#### 4.1.4 Semantic sources

To provide a clean and consistent reference for semantic alignment while avoiding the influence of recognition errors, we use the official 3D semantic annotations provided by the SemanticKITTI labels Behley et al. (2019) as a noise-free source. Each LiDAR point is associated with a semantic class label from the 3D ground truth. During preprocessing, we project these 3D labeled points into the image plane once via the ground-truth extrinsic transformation, producing a fixed image-space probability field P that serves as a stable semantic reference. This projection step is performed only once in the offline phase and does not participate in any optimization. During calibration, the online LiDAR-side distribution $Q_{T}$ is generated from the same 3D labeled points via soft projection under the candidate extrinsic T . Since P is fixed and never updated by the optimizer, and the ground-truth transformation is used only once offline to construct the reference field, this setting constitutes an oracle-style (upper-bound) benchmark. Ground-truth extrinsics are used only for reference construction and evaluation, and are never accessed during the optimization iterations. This deterministic setup isolates calibration behavior from segmentation uncertainty.

#### 4.1.5 Evaluation metrics

Rotation error is defined as the norm of the axis–angle representation ${\left|\log\;\left(R_{\text{est}}R_{\text{gt}}^{-1}\right)\right|}_{2},$ and is reported in degrees. Translation error is computed as $|t_{\text{est}}-t_{\text{gt}}{|}_{2},$ measured in centimeters. In all experiments, rotation is treated as the primary metric for calibration quality: we therefore report mean and median rotation errors, and for the main cross-method comparison (Table 1) and the ablation study (Table 8) we additionally include the maximum rotation error to characterize tail behavior. Translation mainly serves as a sanity check of scale consistency, so we focus on its mean (and, where space allows, median) and omit further statistics to keep the tables compact. Whenever results are pooled across multiple clips, we explicitly state the aggregation scheme.

#### 4.1.6 Efficiency and limitations

Our method targets on-demand/offline calibration with fixed-window batch optimization, using precomputed semantic probability/label maps loaded from disk. Under the paper setting ( N=50 , 376×1241 , K≤40 ), the runtime of our current implementation is minutes-level per window and is dominated by the RGN/LM (semantic I/O <1% ). The current prototype is not optimized for real-time deployment; improving efficiency is left for future work.

### 4.2 Overall comparison across methods

All methods are evaluated on the same 50-frame clips from KITTI sequence 00 with a shared initialization of 5° and 50 mm , following the protocol described in Section 4.1. Robustness to randomized initializations is evaluated separately in Section 4.4.

In addition to our method, we compare against CalibAnything (Luo et al., 2024), DirectVLCalib (Koide et al., 2023), Tsaregorodtsev et al. (2022), and the mutual-information-based method of Pandey et al. (2012), using the publicly available open-source implementations with recommended parameter settings.

Several recent targetless calibration methods also exploit semantic cues, e.g., SemCal (Jiang et al., 2021), SE-Calib (Liao et al., 2023), and SGCalib (Lin et al., 2024). We do not include them in the quantitative comparison in Table 1 because their task settings and evaluation protocols are not directly aligned with our clip-based drift-refinement benchmark, and we could not find publicly available implementations that can be executed under our protocol at the time of revision.

Under this common setting, our method achieves the smallest rotation median and mean among the compared approaches. The reported rotation errors remain at the sub-degree level, whereas the baselines exhibit clearly larger medians and means, especially for Tsaregorodtsev et al., DirectVLCalib and the MI-based method of Pandey et al.

For translation, the mean error of our method is 0.26 cm, while the corresponding values for CalibAnything, DirectVLCalib, Tsaregorodtsev et al., and Pandey et al. (MI) are 12.08 cm, 8.91 cm, 15.85 cm, and 35.74 cm, respectively. These results indicate that, for the chosen dataset and initialization, the proposed semantic distribution alignment with an anchor-fixed sampling measure yields more stable extrinsic estimates in both rotation and translation. The baseline results are based on the authors’ open-source implementations without manual per-clip tuning; although different tuning choices or datasets could change the absolute numbers, the relative performance trends in Table 1 were consistent across all evaluated clips. In addition, this comparison is not fully apples-to-apples: our method currently relies on oracle semantic labels as described in Section 4.1 whereas the baselines operate on their default visual, geometric, or semantic cues, so the numbers in Table 1 should be interpreted as indicative rather than as a definitive ranking. We therefore additionally report a realistic evaluation using network-predicted 2D/3D semantics in Section 4.5.

### 4.3 Cross-sequence generalization

To assess the generalizability of our proposed calibration objective beyond the initially evaluated KITTI odometry sequence 00, we conduct further validation on two additional sequences—KITTI odometry sequences 01 and 07. We follow the same protocol as in Section 4.1: 50-frame clips, the same shared initialization bias (5°/50 mm) , and identical hyperparameters across sequences. Table 2 reports the results.

**TABLE 2:** Cross-sequence generalization of our method on additional KITTI odometry sequences under the shared 5°/50 mm initialization. Rotation errors are in degrees; translation mean errors are in centimeters.

Sequence	Rot. Mean	Rot. Median	Rot. Max	Trans. Mean
01	0.175	0.166	0.524	0.15
07	0.247	0.262	0.348	0.23

Quantitative results indicate that the calibration objective achieves good generalization performance across these sequences. The rotation errors consistently remain at sub-degree magnitudes, with a maximum rotation error of 0.524° observed for sequence 01, while translation errors exhibit stability at the millimeter-to-centimeter scale, showing mean translation errors of 0.15 cm for sequence 01 and 0.23 cm for sequence 07, respectively.

### 4.4 Robustness to random initializations

The deterministic 5°/50 mm perturbation is useful for a controlled cross-method comparison, but it does not characterize how sensitive a solver is to the specific initialization. We therefore additionally evaluate our method under randomized initial perturbations sampled as described in Section 4.1. We run 5 random seeds for each 50-frame clip from KITTI sequence 00 and aggregate the final residual errors after convergence.

In this experiment, we enable the optional coarse hypothesis initialization described in Section 3.6 to obtain a safer starting pose when needed, and then apply the proposed two-stage RGN/LM refinement unchanged. This initializer is executed at the coarsest level and serves only to propose $T_{\text{init}}$ ; it does not modify the objective or the within-stage optimization.

Across all trials, our method converges reliably under both perturbation ranges, achieving sub-degree rotation errors and translation mean errors of 1.60 cm (Moderate) and 2.68 cm (Large). While the proposed solver remains a local optimizer (Section 3.6) and extreme meter-level misalignments may require a coarse global initializer, the above results demonstrate robustness in a practically relevant regime where a rough extrinsic is available. The robustness results are summarized in Table 3.

**TABLE 3:** Robustness of our method to random initialization perturbations on KITTI sequence 00. Moderate: yaw ∈[−10°,10°] , $t_{x,y,z}\in \left[-5,5\right]$ cm; Large: yaw ∈[−20°,20°] , $t_{x,y,z}\in \left[-10,10\right]$ cm. Rotation errors are in degrees; translation errors in centimeters.

Init. Range	Rot. Mean	Rot. Median	Rot. Max	Trans. Mean
Moderate	0.295	0.215	0.991	1.60
Large	0.560	0.615	0.928	2.68

### 4.5 Robustness under network-predicted semantic noise

To evaluate the effect of segmentation noise in a realistic non-oracle scenario, we conduct experiments where both camera and LiDAR semantic fields are derived entirely from neural network predictions. For the camera modality, we employ the InternImage-based 2D semantic segmentation model Wang et al. (2023) with Mask2Former pretrained weights (evaluated via projection of ground-truth LiDAR point clouds, mIoU = 65.85) on each RGB frame to generate per-pixel semantic probability fields P(u) , which represent softmax probabilities. These semantic predictions are computed offline and remain fixed throughout the optimization process. For the LiDAR modality, we obtain per-point semantic predictions using MinkowskiNet (Choy et al., 2019) implemented in OpenPCSeg (official pretrained weights, mIoU = 70.04), and form the projected field $Q_{T}\left(u\right)$ using the same projection operator as in the oracle setting.

All other settings follow Section 4.1. Experimental results obtained from KITTI sequence 07 are summarized in Table 4. As anticipated, replacing oracle annotations with semantic predictions from neural networks introduces additional errors into the calibration results. Despite this increased semantic noise, the optimization framework demonstrates robust and stable convergence under these more practical conditions.

**TABLE 4:** Calibration results under network-predicted 2D/3D semantics on KITTI sequence 07 ( P from InternImage; $Q_{T}$ from MinkowskiNet/OpenPCSeg). Rotation errors are in degrees; translation mean errors are in centimeters.

Setting	Rot. Mean	Rot. Median	Rot. Max	Trans. Mean
InternImage P + MinkowskiNet $Q_{T}$	0.951	1.105	2.752	2.24

### 4.6 Optimizer comparison and convergence behavior

The calibration objective L(T) is optimized over SE(3) and is generally non-convex. In addition to the objective design, the practical outcome can be affected by the numerical optimizer. To isolate the optimizer effect, we keep the objective, initialization (5°/50 mm) , and stopping criteria fixed, and only change the solver.

Our default solver is a Riemannian Gauss–Newton method with Levenberg–Marquardt damping (RGN/LM) (Section 3.6). As a first-order baseline, we implement Riemannian gradient descent (RGD) with Armijo backtracking line search. Table 5 summarizes the final calibration errors and iteration statistics aggregated over representative clips from KITTI sequence 00. Under the same experimental setting, RGN/LM reaches substantially lower final rotation and translation errors than RGD.

**TABLE 5:** Optimizer comparison aggregated over KITTI sequence 00 clips under the shared 5°/50 mm initialization. We report mean across clips. Rotation errors are in degrees; translation errors are in centimeters.

Optimizer	Rot. Mean	Trans. Mean	Outer iters. Mean
RGN/LM (ours)	0.188	0.26	5
RGD + Armijo	0.362	1.29	6.8

The reported iteration count is provided only as a coarse convergence statistic and should not be interpreted as computational cost. In particular, Armijo backtracking may evaluate the objective multiple times within a single RGD iteration, whereas LM damping changes the effective step acceptance and progress per iteration. Accordingly, we interpret the comparison primarily through the final accuracy, and use the iteration numbers only as auxiliary information.

### 4.7 Effect of semantic class-distribution shifts

To examine how differences in scene composition affect calibration performance, we characterize each 50-frame clip by the pixel-level proportions of *road* and *vehicle* labels in P (road versus non-road/vehicle), and group clips from KITTI sequence 00 according to these statistics.

For this analysis, we use an extended set of 18 non-overlapping 50-frame clips from sequence 00, starting at frames 0, 200, 400, 600, 800, 1,000, 1,200, 1,400, 2000, 2,200, 2,400, 2,600, 2,800, 3,000, 3,200, 3,400, 3,600, and 3,800. Based on their measured road/vehicle pixel proportions, these clips are partitioned into three semantic categories:

- A1 Road-dominant: clips in which the roadway accounts for the largest share of pixels and vehicles are comparatively rare. This group contains 00_s0000, 00_s0400, 00_s1200, 00_s1400, 00_s3000, and 00_s3400.
- A2 Vehicle-dense: clips with the strongest vehicle and non-road coverage. This group contains 00_s0600, 00_s1000, 00_s2200, 00_s2800, 00_s3200, and 00_s3600.
- A3 Balanced: the remaining clips. This group contains 00_s0200, 00_s0800, 00_s2000, 00_s2400, 00_s2600, and 00_s3800.

All experiments in this subsection use the shared deterministic initialization bias (5°/50 mm). For each clip, we run the calibration once under this fixed perturbation and aggregate the resulting residuals over all clips within each cluster.

As summarized in Table 6, A2 and A3 achieve similarly low mean rotation residuals (around 0.19° ), indicating that the proposed method remains stable in both balanced and vehicle-rich scenes. In the vehicle-dense A2 group, the median rotation error further drops to about 0.110° , which is consistent with the additional geometric support provided by vehicles and other non-road structures.

**TABLE 6:** Calibration residuals across semantic scene categories on KITTI sequence 00 (18 clips in total, 6 clips per category). Rotation errors are reported in degrees; translation errors in centimeters.

Scene category	Rot. Mean	Rot. Median	Trans. Mean
A1 road-dominant	0.333	0.102	0.40
A2 vehicle-dense	0.185	0.110	0.24
A3 balanced	0.191	0.196	0.22

The behavior of A1 is noticeably different. Although the median rotation error remains small (around 0.102° ), the mean increases to 0.333° and the average translation residual also becomes larger. This indicates that occasional larger errors occur more frequently in road-dominant scenes, where vertical structures are scarce and yaw is more easily coupled with lateral translation.

Overall, these results suggest that the scene’s semantic composition has a clear impact on calibration quality. Scenes with richer structure (A2, A3) tend to produce more stable estimates, whereas road-dominant segments (A1) naturally exhibit weaker observability. A practical implication is that LiDAR–camera recalibration does not need to run continuously: it is more effective to trigger recalibration only on clips that contain sufficient semantic and structural richness (similar to A2 or A3), while largely ignoring frames dominated by road surface, thereby reducing computational load without degrading calibration quality. We operationalize this intuition with the following lightweight triggering heuristic.

Motivated by the above finding that road-dominant segments exhibit weaker observability, we propose a simple online triggering rule for practical deployment. Given per-frame semantic probability maps $P_{k}$ , we maintain sliding-window estimates of the road and vehicle pixel proportions $\left(\rho_{\text{road}}\left(k\right),\rho_{\text{veh}}\left(k\right)\right)$ over the most recent L frames using the same definitions as in this subsection. We trigger recalibration when the window is not road-dominant and contains sufficient non-road structure, as summarized in Equation 28:

$$\rho_{\text{road}}\left(k\right)\leq \tau_{\text{road}},\;\rho_{\text{veh}}\left(k\right)\geq \tau_{\text{veh}},\;N_{\text{proj}}\left(k\right)\geq N_{\min},$$ (28)

where $N_{\text{proj}}\left(k\right)$ denotes the number of valid projected LiDAR points within the image. The thresholds $\left(\tau_{\text{road}},\tau_{\text{veh}}\right)$ can be set automatically from running statistics (e.g., percentile-based filtering) to suppress road-dominant windows. Note that this heuristic is proposed for online triggering and is not used to select the fixed evaluation clips in this subsection.

### 4.8 Effect of semantic mass prior and gating

We next examine how the semantic mass prior and its gating mechanism influence yaw observability and the stability of translation estimates. On top of the default configuration, we consider three variants of the semantic weighting:

- M1 Vehicle-tuned (strict): a prior originally tuned for vehicle-dense scenes, using a higher non-road gain and a relatively selective retention of pixels.
- M2 Mass-loose (relaxed): a relaxed prior with a lower non-road gain and a less selective retention, admitting more low-mass (low-support) pixels into the loss.
- M3 Mass-strict (tightened): an over-pruned prior that keeps only a small subset of relatively “clean” non-road pixels.

All three settings are evaluated on the same clip set and with the same fixed initial perturbation, and the residuals are aggregated over all clips.

As shown in Table 7, the vehicle-tuned setting M1 achieves the lowest median rotation error (0.110°) and the smallest mean translation error (0.24 cm). In this regime, discarding obviously noisy pixels while still preserving sufficient non-road coverage improves the effective signal-to-noise ratio and leads to stable rotation and translation estimates.

**TABLE 7:** Sensitivity to the semantic mass prior and gating (rotation in degrees, translation in centimeters).

Configuration	Rot. Mean	Rot. Median	Trans. Mean
M1 vehicle-tuned (strict)	0.185	0.110	0.24
M2 Mass-loose (relaxed)	0.188	0.199	0.35
M3 Mass-strict (tightened)	0.275	0.201	0.45

When the prior is relaxed (M2), the rotation statistics remain close to those of M1, but the translation mean increases to 0.35 cm. Admitting more low-mass (low-support) pixels does not immediately harm rotation accuracy, but it can reduce translation stability, as reflected by the higher mean translation error.

If the prior is tightened too aggressively (M3), both rotation and translation degrade: the mean rotation error rises to 0.275° , and the mean translation error increases to 0.45 cm. In this case, the pixel set is pruned so strongly that the objective loses curvature in the rotation direction.

Taken together, these variants and the scene-type analysis in Section 4.7 suggest a simple guideline: use a moderately conservative prior by default, tighten it only in clearly vehicle-dense scenes, and keep it less aggressive in road-dominant segments.

### 4.9 Ablation analysis

Table 8 summarizes the effect of several key components, with rotation errors reported in degrees and translation errors in centimeters.

**TABLE 8:** Ablation results. Rotation in degrees; translation in centimeters.

Ablation	Rot. Mean	Rot. Median	Rot. Max	Trans. Mean
Baseline (full)	0.188	0.198	0.500	0.26
Anchor-fixed measure → dynamic	0.252	0.209	0.670	0.41
Continuous field → discrete label	0.471	0.301	1.100	0.83
Multi-scale → single-scale (1.0 only)	0.219	0.203	0.587	0.33
Kernel bandwidth σ=0.5×	0.198	0.199	0.548	0.27
Kernel bandwidth σ=2.0×	0.203	0.200	0.556	0.37
Directional observability weighting off	0.231	0.208	0.598	0.28
JS risk $\to L_{2}$	0.316	0.234	0.742	0.35
Histogram matching removed	0.261	0.211	0.603	0.33

Replacing the bounded JS risk with a plain $L_{2}$ loss yields the worst overall performance. The mean rotation error increases from 0.188 to 0.316° , the translation mean rises from 0.26 to 0.35 cm, and the maximum rotation error grows from 0.500° to 0.742° . In this configuration, a few large residuals in outlier/mismatch regions dominate the $L_{2}$ objective, showing that the JS-based formulation is critical for keeping tail errors in check.

Disabling the directional observability weighting weakens the contribution of yaw-sensitive pixels and effectively shrinks the update step along the yaw axis. The mean rotation error increases by about 23% (0.188 → 0.231), and the translation mean also deteriorates (0.26 → 0.28 cm). Emphasizing yaw-sensitive pixels is important for both convergence stability.

Removing the global histogram term produces errors between the two extremes above, but still increases the maximum rotation error to 0.603° and raises the translation mean to 0.33 cm. Without this global class-distribution constraint, road pixels tend to dominate the measure in some clips, making the method more vulnerable to long-tail failures in regions where semantic evidence is weak.

From a system perspective, these ablations suggest a clear division of labor: the JS loss handles outliers, the observability weighting stabilizes yaw, and the histogram matching corrects for semantic bias in road-heavy scenes. The full configuration in Table 8 combines these effects and explains the compact error distributions observed in the main results.

Beyond the core JS risk and yaw-weighting, Table 8 also validates several implementation choices. (i) Anchor-fixed → dynamic measure recomputes the sampling/weighting measure at every iteration, which reintroduces a moving reference and degrades accuracy, supporting our anchor-fixed design. (ii) Continuous field → discrete label replaces the probabilistic semantic field with hard labels, reducing smoothness of the objective and harming convergence. (iii) Multi-scale → single-scale removes the coarse-to-fine support and leads to a consistent performance drop, indicating the benefit of multi-scale alignment. (iv) A light kernel bandwidth sweep shows the method is reasonably stable around the default σ ; overly small/large σ tends to under/over-smooth the field and slightly worsens the solution.

This confirms that emphasizing yaw-sensitive pixels is important for stable optimization and yaw identifiability. A 1D objective slice along yaw is provided in Supplementary Figure S1 to visualize the local optimization behavior of our objective.

### 4.10 Qualitative comparison

**FIGURE 2:**
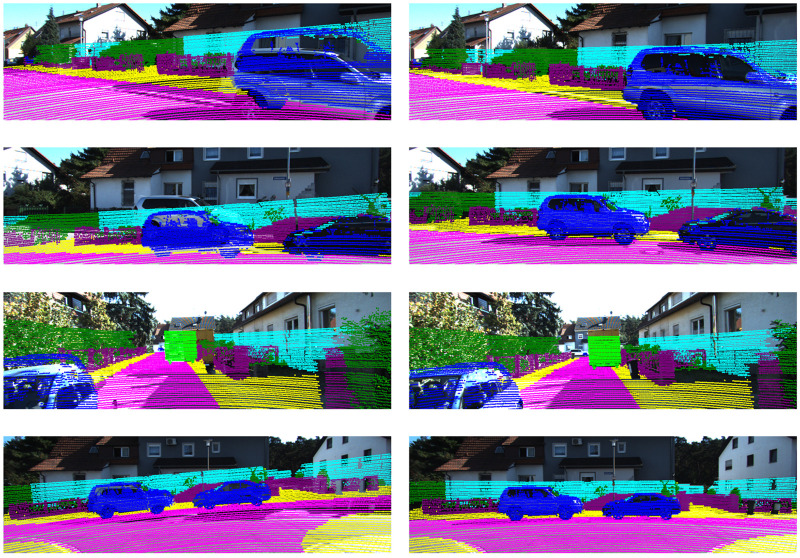
Qualitative comparison before (left) and after (right) calibration. After optimization, the projected LiDAR points align closely with image structures such as road edges, vehicle contours and building facades.

Figure 2 presents a qualitative comparison illustrating the calibration effect under a representative initial drift, showing how the proposed method corrects the misalignment between the two modalities. Before calibration, noticeable misalignments and parallax can be observed between the LiDAR projection and scene structures such as building facades, guardrails and vehicle contours, revealing the underlying extrinsic errors between the sensor coordinate frames. After optimization, the projected point cloud aligns closely with these structures at the pixel level: road edges exhibit continuous and well-fitted point distributions, vertical edges of building facades remain consistent across the two modalities, and vehicle outlines match the point cloud boundaries with high fidelity.

These qualitative observations are consistent with the quantitative statistics and the semantic-cluster analyses in Section 4, and visually corroborate the sub-degree rotation and millimeter-level translation residuals reported in our results.

## 5 Discussion

This work introduces a semantic distribution alignment method that directly targets the metric drift often observed in LiDAR–camera calibration. The anchor-fixed sampling measure keeps the optimization metric stable, while the direction-aware weighting is designed to improve yaw identifiability in scenes with limited geometric cues; this design is reflected in the reduced rotation errors observed in our ablations. The log-saturated JS divergence, together with semantic histogram balancing and multi-scale processing, further improves robustness to outliers and cross-modal mismatches and to sparse semantic support. Experiments on the KITTI odometry show that the method achieves higher accuracy and stronger robustness than the representative geometric, semantic-mask, and direct-alignment baselines reproduced in our experiments. Ablation studies confirm that the observability weighting, the JS-based risk, and the global histogram term each contribute independently and complementarily, while the anchor-fixed measure stabilizes the metric by construction.

The method still faces challenges in cases with extremely sparse semantics or large cross-modal discrepancies. Translation observability may also degrade during long periods of weak excitation, such as extended straight-line motion, and incorporating additional geometric or semantic cues may help address these limitations. In this work, we primarily benchmark on KITTI odometry sequence 00 and additionally evaluate on sequences 01 and 07 (Section 4.3) with clean SemanticKITTI labels in order to isolate calibration behavior from segmentation uncertainty. We further report a realistic evaluation under network-predicted 2D/3D semantics in Section 4.5. Extending the evaluation to the full KITTI odometry set and to additional datasets remains an interesting direction for future work and would further clarify robustness under realistic perception pipelines.

## 6 Conclusion

This work introduced a LiDAR–camera extrinsic calibration framework based on semantic distribution alignment. By combining an anchor-fixed sampling measure with direction-aware weighting and a robust JS-based semantic objective, the method achieves stable optimization and low residual errors under both the shared deterministic 5°/50 mm initialization used for cross-method comparison and randomized initial perturbations up to ±20° yaw and ±10 cm per-axis translation. In addition to the main benchmark on KITTI sequence 00, cross-sequence experiments on KITTI sequences 01 and 07 further suggest that the proposed objective generalizes beyond a single sequence. Beyond this setting, future work will extend the framework to multi-frame optimization with temporal consistency, incorporate explicit occlusion handling, and evaluate the approach on additional datasets.

## Data Availability

Publicly available datasets were analyzed in this study. The KITTI Odometry dataset can be found at http://www.cvlibs.net/datasets/kitti/, and the SemanticKITTI labels can be found at http://www.semantic-kitti.org/.
