# Supplementary material for: Targetless LiDAR–camera extrinsic calibration via semantic distribution alignment
Source: Front Robot AI. 2026 Mar 9;13:1760867. doi: 10.3389/frobt.2026.1760867 (PMC13006848; doi:10.3389/frobt.2026.1760867)
Supplement: Supplementary file 1 [file Image1.pdf]

Supplementary Material

Targetless LiDAR–Camera Extrinsic Calibration via Semantic Distribution Alignment

S1. 1D yaw objective slice

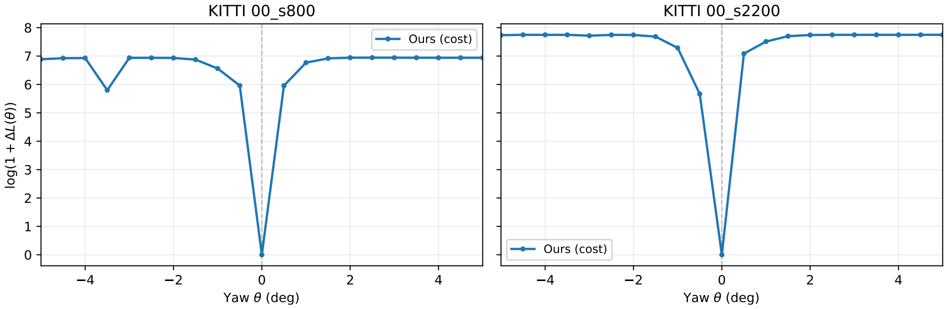

Figure 1: 1D objective slice along yaw on representative KITTI clips (00_s800 and 00_s2200, nf=50). We sweep a yaw perturbation θ around a reference extrinsic T_0 and evaluate the optimized objective $\mathcal{L}(T(\theta))$. The y-axis shows $\log(1 + \Delta\mathcal{L}(\theta))$, where $\Delta\mathcal{L}(\theta) = \mathcal{L}(T(\theta)) - \min_\theta \mathcal{L}(T(\theta))$. Here $\theta = 0^\circ$ corresponds to T_0. This is a local 1D diagnostic slice and does not imply global convexity.
